# Leveraging machine learning to identify determinants of zero utilization of maternal continuum of care in Ethiopia: Insights from SHAP analysis and the 2019 mini DHS

**DOI:** 10.1371/journal.pgph.0004787

**Published:** 2025-06-20

**Authors:** Shimels Derso Kebede, Agmasie Damtew Walle, Daniel Niguse Mamo, Ermias Bekele Enyew, Jibril Bashir Adem, Meron Asmamaw Alemayehu

**Affiliations:** 1 Department of Health Informatics, School of Public Health, College of Medicine and Health Science, Wollo University, Dessie, Ethiopia; 2 Department of Health Informatics, School of Public Health, Asrat Woldeyes Health Science Campus, Debre Birhan University, Debre Berhan, Ethiopia; 3 Department of Health Informatics, College of Medicine and Health Sciences, Arba Minch University, Arba Minch, Ethiopia; 4 Department of Public Health, College of Medicine and Health Science, Arsi University, Asella, Ethiopia; 5 Department of Epidemiology and Biostatistics, Institute of Public Health, College of Medicine and Health Sciences, University of Gondar, Gondar, Ethiopia; PLOS: Public Library of Science, UNITED STATES OF AMERICA

## Abstract

Ensuring complete utilization of maternal continuum of care is essential for reducing maternal and neonatal mortality. In Ethiopia, significant gaps remain in maternal healthcare utilization, particularly among women who do not engage in any stage of the maternal care continuum. This study aims to identify the determinants of zero utilization in the maternal continuum of care among Ethiopian women using machine learning techniques, with insights provided by SHAP (SHapley Additive exPlanations) analysis. This study analyzed data from the 2019 Ethiopian Mini Demographic and Health Survey, using a cross-sectional design. The dataset was preprocessed and modeled using various machine learning algorithms through the PyCaret library, with lightGBM emerging as the best model after various models trained and evaluated based on classification performance metrics. S Synthetic Minority Over-sampling Technique was applied to address class imbalance. SHAP analysis was used to interpret model predictions and identify key predictors. lightGBM demonstrated robust performance with an accuracy of 84.47%, an AUC of 0.93, a recall of 0.80, a precision of 0.95, and an F1-score of 0.87 on test data. SHAP analysis revealed that residence in rural areas, the Somali region, being a daughter in the household, and Protestant religion were positively associated with zero maternal care utilization. Conversely, secondary or higher education, being married, higher wealth status, and having multiple children were associated with lower likelihoods of zero care utilization. The findings highlight the critical role of socioeconomic, demographic, and regional factors in maternal care utilization in Ethiopia. Targeted interventions, particularly in rural and underserved areas, are necessary to reduce barriers and promote equitable access to maternal healthcare services across Ethiopia. These insights can inform policies aimed at expanding female education, strengthening community-based maternal health programs, and prioritizing resource allocation to regions such as Somali where zero utilization is highest.

## Introduction

Sustainable Development Goal 3 (SDG 3) aims to ensure healthy lives and promote well-being for all, with a specific target to reduce the global maternal mortality. Achieving this goal requires a focus on improving access to comprehensive maternal healthcare services, including antenatal, delivery, and postnatal care, which form the core of the maternal continuum of care. Access to these services is critical determinant of maternal and neonatal health outcomes globally. It is vital for reducing preventable maternal and perinatal mortality, ensuring comprehensive healthcare access throughout pregnancy, childbirth, and postpartum periods [1,2].

Sub-Saharan Africa has a disproportionately high burden of maternal mortality, significantly contributing to global maternal death statistics [3]. Maternal mortality in Sub-Saharan Africa is a leading preventable cause of death among adults, significantly higher than global averages [4], reflecting severe health system challenges in the region. Inadequate access to maternal healthcare services remains a pressing issue that calls for a better understanding of the underlying barriers preventing women from receiving continuous care.

In Ethiopia, maternal healthcare services have shown improvements in recent years, yet significant gaps remain. According to the Ethiopian Mini Demographic and Health Survey (EMDHS) 2019, 74% of women aged 15–49 who had a live birth in the five years preceding the survey received antenatal care from a skilled provider for their most recent birth, but only 43% attended the recommended four or more visits during their last pregnancy [5]. Furthermore, postnatal care coverage remains low, with just 34% of women and 35% of newborns receiving a check-up within the crucial first two days after birth. These statistics underscore the ongoing challenges in ensuring a full continuum of maternal care and highlight the issue of women who receive no maternal healthcare services.

Zero utilization of the maternal continuum of care has significant clinical consequences for both mothers and newborns. Women who forgo antenatal, delivery, and postnatal care face a significantly higher risk of maternal mortality and morbidity, including complications that can lead to long-term disability [6]. For newborns, the absence of maternal healthcare contributes to elevated neonatal mortality and a higher likelihood of preventable deaths that could have been mitigated through timely medical intervention [6,7]. These outcomes highlight the importance of ensuring that all women, particularly those currently receiving no care, are integrated into the healthcare system to improve survival and well-being for both mothers and their infants.

Recent studies have investigated factors influencing maternal healthcare utilization in Ethiopia, with a focus on social, economic, and geographic determinants [8–11]. While previous studies on maternal healthcare utilization in Ethiopia have largely focused on either individual service or the completion of the maternal continuum of care—covering antenatal care (ANC), skilled delivery, and postnatal care (PNC)—they tend to overlook women who receive no care at all across these stages. Although a previous study [12] has explored predictors of zero maternal care utilization in Ethiopia, our study provides a unique contribution by using the most recent 2019 Ethiopian Mini DHS data—the first dataset collected after the implementation of Ethiopia’s Health Sector Transformation Plan I (HSTP-I). This allows us to assess the current state of maternal continuum of care utilization in the context of recent national health initiatives aimed at expanding access and improving maternal health outcomes. Furthermore, most research employs classical statistical approaches, such as linear and logistic regression, to identify factors associated with partial or complete care utilization, but these approaches often fail to capture the complex dynamics leading to zero maternal continuum of care. This gap highlights the need for more sophisticated methods, such as machine learning, which can better detect hidden patterns and non-linear relationships that classical approaches might miss. By addressing this overlooked phenomenon, this study aims to provide new insights into the determinants of non-utilization of maternal healthcare services.

Given the potential of machine learning to advance healthcare analytics, this research will provide valuable insights into maternal healthcare utilization in Ethiopia. These findings can guide the development of more effective interventions to ensure women receive the full continuum of maternal care. Hence this study aimed at using machine learning to identify the determinants of zero maternal continuum of care among reproductive-age women in Ethiopia using a nationally representative data. Furthermore, by capturing updated trends and outcomes, our study offers timely insights into the effectiveness of HSTP-I interventions and highlights areas where further progress is needed. Understanding the key determinants of zero maternal continuum of care in Ethiopia is essential for improving maternal and child health outcomes. By identifying the factors contributing to low service uptake, targeted interventions can be designed to address the gaps in maternal healthcare.

## Methods

### Study design and data source

This study utilized a cross-sectional design based on data from the 2019 Ethiopian Mini Demographic and Health Survey, a nationally representative survey conducted to collect key health indicators, including maternal and child health. The survey data were collected using a two-stage sampling procedure, ensuring coverage of both urban and rural areas across Ethiopia’s 11 regions from March 21, 2019, to June 28, 2019 [5]. This survey offers comprehensive information on the use of maternal health services, which is critical for understanding the factors associated with zero maternal continuum of care among women of reproductive age.

### Study population and sample

The study’s source population included all women aged 15–49 who had given birth in the five years preceding the data collection period of the 2019 Ethiopian Mini Demographic and Health Survey. From this source population, the study focused on women residing in the selected enumeration areas (EAs) during the same period, making them the study population.

A total weighted sample of 6,913 women who met the criteria was used in the final analysis. This sample, which was adjusted for the survey’s complex sampling design, provides nationally and regionally representative estimates for maternal healthcare service utilization across Ethiopia.

### Study variables

The outcome variable for this study is zero maternal continuum of care, defined as the failure to receive all three maternal healthcare services: antenatal care (ANC), skilled delivery, and postnatal care (PNC) within 48 hours [12]. This binary variable is coded as ‘1’ for women who did not receive any of these services during pregnancy, childbirth, or postpartum, and ‘0’ for women who accessed at least one of these services. Predictor variables used in this analysis include a range of socio-demographic, economic, and health-related factors such as maternal age, educational level, wealth index, residence, region, sex of household head, birth order, marital status, religion, household size, relation with household head, and number of children (parity). These variables were selected based on their documented importance in influencing maternal healthcare utilization and their availability in the EMDHS dataset.

### Data management and analysis

The dataset from the 2019 EMDHS was initially obtained in Stata format (.dta) and imported into the R environment for data cleaning and preprocessing. Any missing data were handled using the Multivariate Imputation by Chained Equations (MICE) package in R, which imputes missing values based on the observed data distribution. This process ensured that the imputed values reflected the underlying patterns within the data, reducing bias and maintaining statistical validity. After the imputation, the complete dataset was exported in CSV format for the machine learning analysis in Python.

Further data preprocessing such as categorical encoding and one-hot encoding of categorical variables was done in python prior to model training. Furthermore, the data underwent stratified splitting into 80% training and 20% test set to maintain the class distribution in both the training and test sets. For model building and prediction, the PyCaret library [13] was used. In PyCaret, several popular machine learning models are available for classification tasks, which are fitted and compared during the model-building process. For this study, a variety of linear and non-linear algorithms such as K Nearest Neighbors (KNN), Naïve Bayes, multilayer perceptron (MLP), Ridge regression, eXtreme gradient boosting (XGBoost), Light Gradient Boosting Machine (lightGBM), Random Forest, and Support Vector Machine (SVM) were trained to predict zero maternal continuum of care utilization. A 10-fold cross-validation (CV) technique was employed to develop and evaluate performance of selected models. This approach enhances the generalizability of the models by splitting the data into 10 parts, training on nine parts, and testing on the remaining part in each iteration. The average performance across all folds was used to assess the models. These models were evaluated based on performance metrics such as accuracy, precision, recall, and the area under the receiver operating characteristic curve (AUC). The best-performing model was selected based on cross-validation results, ensuring robustness and generalizability to unseen data.

Although the degree of class imbalance was relatively mild [14], with class 1(zero maternal continuum of care) comprising 63.65% of cases and class 0 making up 36.35%, steps were taken to mitigate any potential bias. During model training, PyCaret’s built-in SMOTE (Synthetic Minority Over-sampling Technique) was applied within the cross-validation process. This approach slightly augmented the minority class by generating synthetic samples, allowed the model to more effectively identify patterns across both classes, balancing accuracy and sensitivity without compromising the original data distribution.

Finally, SHapley Additive exPlanations (SHAP) values were employed to interpret the model’s predictions and understand the role of individual variables. SHAP provides insights into how each feature contributes to the model’s decision-making process, enabling a transparent and interpretable machine learning solution. Visualizations such as summary plots, beeswarm plots and waterfall plots were generated to depict the contribution of these features, enhancing the interpretability of the findings.

### Ethics approval and consent to participate

Permission to use the data has been granted by the Measure DHS program through legal registration. Mini EDHS (2019) data was used which is available on the public domain through the Measure DHS website (www.measuredhs.com). Consent for participation is not applicable for this study since secondary data was used for the analysis. Furthermore, the DHS survey takes informed consent of participation from the survey participants.

## Results

### Socio-demographic and economic characteristics of study participants

A total weighted sample of 6913 reproductive-age women were included in the study. Among these sample, majority of women resided in rural areas, with 68.09% living in rural locations and only 31.91% in urban areas (Table 1). In terms of marital status, the largest proportion of women were married (62.20%), followed by 33.18% who were single, while smaller percentages were either divorced (3.91%) or widowed (0.72%). Regarding educational attainment, 44.87% of the women had primary education, 34.01% had no formal education, and 21.13% had completed secondary or higher education.

**Table 1:** Characteristics of study participants (n = 6913), EMDHS 2019.

Variable	Category	Frequency	Percentage
place of residence	Urban	2206	31.91
	Rural	4707	68.09
marital status	Single	2293	33.18
	Married	4300	62.20
	Widowed	50	0.72
	Divorced	270	3.91
highest educational level	no education	2351	34.01
	Primary	3102	44.87
	secondary or higher	1460	21.13
Religion	Orthodox	2808	40.62
	Protestant	1874	27.11
	Muslim	2116	30.60
	Others^*^	115	1.66
Region	Tigray	490	7.09
	Afar	69	1.00
	Amhara	1516	21.92
	Oromia	2626	37.99
	Somali	356	5.14
	Benishangul Gumuz	76	1.10
	SNNPR	1313	19.00
	Gambela	30	0.44
	Harari	21	0.30
	Addis Ababa	366	5.30
	Dire Dawa	50	0.72
Woman’s age	15–24	3620	52.37
	25–34	2290	33.12
	35–49	1003	14.51
relationship to household head	Head	593	8.58
	Wife	3498	50.60
	Daughter	2003	28.97
	Other	819	11.85
sex of household head	Male	5591	80.88
	Female	1322	19.12
Birth order	<=3	2078	53.51
	>3	1805	46.49
Wealth status	Poor	2476	35.82
	Rich	3155	45.64
	Middle	1282	18.55
number of household members	1–4	2427	35.11
	5–9	4019	58.14
	10 or more	467	6.75
number of living children (Parity)	Zero	3069	44.39
	1–4	2683	38.82
	5 or more	1161	16.79

* others = catholic, traditional and other unspecified religions.

Regionally, the largest number of respondents came from Oromia (37.99%) and Amhara (21.92%), while other regions like Somali (5.14%), SNNPR (19.00%), and Tigray (7.09%) had smaller representation. Women from urban regions like Addis Ababa represented 5.30% of the total sample. In terms of religion, 40.62% of the participants were Orthodox Christians, followed by 30.60% Muslims and 27.11% Protestants. The age distribution indicated that 52.37% of the women were between 15 and 24 years old, followed by 33.12% between 25 and 34 years, and 14.51% aged 35–49 years. The majority of respondents were wives (50.60%) in their households, while 28.97% were daughters, and 8.58% were household heads. Most of the households were headed by men (80.88%), and 19.12% were headed by women.

Household size varied, with 58.14% of the respondents living in households with 5–9 members, 35.11% in households with 1–4 members, and only 6.75% in households with 10 or more members. Regarding parity, 44.39% of women had no living children, while 38.82% had 1–4 children, and 16.79% had 5 or more children. In terms of wealth status, 45.64% of the women were classified as rich, 35.82% as poor, and 18.55% as middle-income.

### Performance comparison of trained models

The results of this study, derived from 10-fold cross-validation on the training data, show that several machine learning models demonstrated robust classification abilities on key performance metrics. Among the models tested, the lightGBM achieved the highest overall performance, with an accuracy of 83.74% and an area under the curve (AUC) of 0.93. The lightGBM also exhibited strong recall (0.83) and precision (0.91), leading to an F1 score of 0.87 (Table 2). The remaining models, including the MLP, KNN and Naive Bayes, displayed lower performance metrics, with Naive Bayes achieving the lowest accuracy (73.73%) and AUC (0.83).

**Table 2:** Performance of trained models.

Model	Accuracy (%)	AUC	Recall	Precision	F1
KNN	80.53	0.89	0.81	0.87	0.84
Naive Bayes	73.73	0.83	0.69	0.88	0.77
MLP Classifier	82.10	0.92	0.82	0.89	0.85
Ridge Classifier	82.56	0.93	0.75	0.97	0.84
Random Forest	82.92	0.91	0.85	0.88	0.86
XGBoost	83.41	0.92	0.84	0.89	0.86
SVM	83.69	0.93	0.79	0.94	0.86
lightGBM	83.74	0.93	0.83	0.91	0.87

While some models had marginally better individual metrics, LightGBM provided higher accuracy, AUC and the best trade-off between precision and recall by maximizing f1-score, which is critical for handling class imbalances and ensuring reliable predictions. Overall, LightGBM was selected as the final model due to its balanced performance across all evaluation metrics, making it the most suitable choice for predicting zero maternal continuum of care.

**Fig 1:**
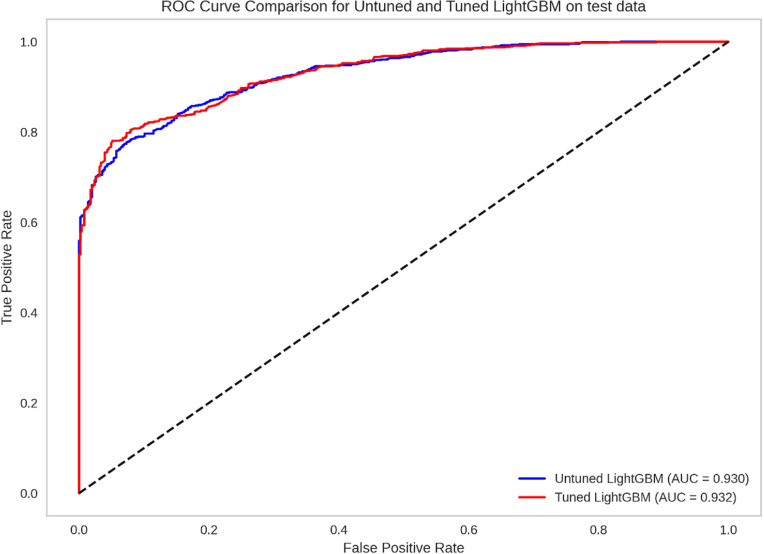
ROC Curve showing the performance of tuned and un-tuned lightGBM model on the test data.

**Fig 2:**
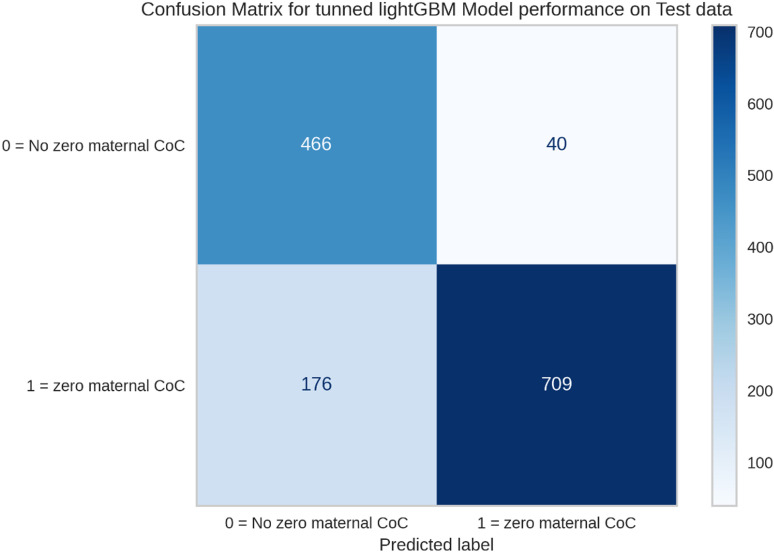
Confusion matrix for prediction of tuned lightGBM model on the test data.

Following model selection, LightGBM was further optimized through PyCaret’s hyperparameter tuning method, which enhanced its predictive capacity (Table 3). The optimized LightGBM model was then applied to previously unseen test data to evaluate its final performance. The model achieved an accuracy of 84.47%, an AUC of 0.93 (Figs 1 and 2), recall of 0.80, precision of 0.95, and an F1-score of 0.87. These results indicate that the tuned LightGBM model effectively balanced recall and precision, supporting its robustness for this classification task.

**Table 3:** LightGBM hyperparameters with default values, search ranges, and selected optimal values.

Parameter Name	Default Value	Range Used	Optimal Value
num_leaves	31	20 – 150	70
max_depth	-1 (no limit)	0 – 15	8
learning_rate	0.1	0.01 – 0.3	0.05
n_estimators	100	50 – 150	80
reg_alpha (L1)	0	0 – 1	0.1
reg_lambda (L2)	0	0 – 1	0.2

The confusion matrix presented in our study provides a comprehensive overview of the model’s performance in predicting the presence or absence of a zero maternal continuum of care on the test data. The matrix reveals that out of a total of 1,391 instances, the model correctly identified 709 cases as having a zero maternal continuum of care, which are classified as true positives. Additionally, it accurately predicted 466 instances as not having a zero maternal continuum of care, categorized as true negatives. However, the model also misclassified 40 instances as having a zero maternal continuum of care when they did not, resulting in false positives, and it failed to identify 176 instances that actually had a zero maternal continuum of care, which are classified as false negatives (Fig 2). This indicates the model’s strong ability to distinguish between women who did and did not receive the continuum of maternal care, making it a reliable tool for predicting and potentially informing interventions in maternal healthcare.

### Predictors of zero maternal continuum of care

**Fig 3:**
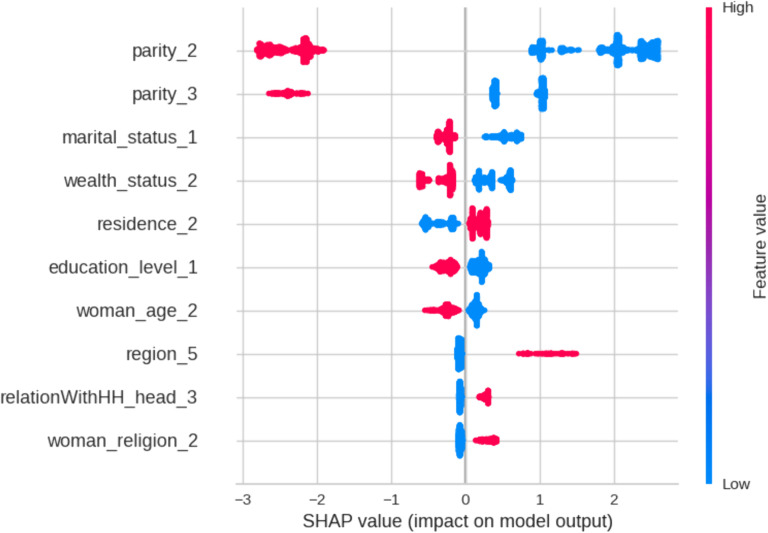
SHAP summary plot of the top predictor variables for zero maternal continuum of care.

The SHAP beeswarm summary plot in Fig 3 serves as a valuable feature selection tool, identifying the top predictor variables for zero maternal continuum of care among women in Ethiopia. This visualization not only highlights the most influential features but also clarifies how each one contributes to the outcome variable. Each predictor is a binary categorical variable, coded as either 1 or 0, representing specific demographic and socioeconomic characteristics. This plot allows us to examine the relative importance of each feature and its direction of impact, whether it increases or decreases the likelihood of zero maternal care.

Accordingly, parity, marital status, wealth status, place of residence, education level of the woman, age of the woman, administrative region, relationship of the woman with households head, and woman religion were top predictors of for zero maternal continuum of care. The analysis revealed that several factors were positively associated with zero utilization of maternal continuum of care. Women residing in rural areas (residence_2) had a higher likelihood of zero maternal care utilization, as did women from the Somali region (region_5) (Fig 3). Additionally, daughters of household heads (relationWithHH_head_3) and Protestant women (woman_religion_2) were more likely to experience zero utilization of maternal continuum of care. The SHAP result also indicated that certain features were associated with a reduced likelihood of zero utilization of maternal continuum of care. Women with one to four living children (parity_2) and those with five or more living children (parity_3) had a lower risk of zero maternal care utilization. Similarly, married women (marital_status_1), those in the higher wealth category (wealth_status_2), women aged 25–34 years (woman_age_2), and those with secondary or higher education (education_level_1) were less likely to experience zero utilization of maternal continuum of care.

## Discussion

This study provides a comprehensive analysis of the predictors associated with zero utilization of maternal continuum of care among Ethiopian women, using a robust machine learning approach to identify the most influential socioeconomic and demographic factors. Accordingly, the final model, lightGBM, demonstrated strong performance on the test data, achieving an accuracy of 84.47%, an AUC of 0.9321, a recall of 80.11%, a precision of 94.66%, and an F1-score of 86.78%. These metrics indicate a reliable model that effectively discriminates between women who are likely and unlikely to utilize maternal care services, enhancing our understanding of the underlying patterns in maternal health-seeking behavior.

Through interpretable machine learning analysis, several predictors emerged as significant, with distinct patterns observed in both positive and negative associations with zero maternal continuum of care. Consequently, factors such as parity, marital status, wealth status, place of residence, educational attainment of the woman, age of the woman, region, and the woman’s relationship with the head of the household, and religion of the woman emerged as the influential predictors of a complete absence of maternal continuum of care.

Women residing in rural areas had a higher likelihood of zero maternal continuum of care utilization, supporting literature on geographic disparities in maternal healthcare access across Ethiopia [15–17]. This finding was also in line with a study conducted across 88 countries, which reported rural women have greater challenges in prenatal, delivery, and postnatal care [18]. This relationship could be explained by limited healthcare service availability and accessibility in rural areas. Women in rural areas face challenging physical access to health facilities as longer distance from health facility [12,19] and limited physical accessibility [20] have a clear implication for non-utilization of maternal healthcare services. Compounding this, rural health facilities often lack skilled providers and focus on preventive services, leaving comprehensive maternal care underserved [21]. Additionally, women reside in the Somali region was associated with a higher likelihood of zero maternal care. This finding aligns with previous study done in one of the region’s administrative zones reported only 13.5% of mothers received all essential standard maternal continuum of care [22]. Other studies also reported similar finding of lower maternal service utilization in the region [23,24]. This regional disparity may reflect distinct socioeconomic and cultural factors that influence healthcare access, such as lower service availability, regional variations in health-seeking behavior, and differing levels of community-based health services. Another possible explanation for this could be that Somali is one of the country’s pastoralist regions, where the nomadic lifestyle and geographic mobility often complicate consistent healthcare access in the region.

Multiparous women who have between one and four living children, as well as those who have five or more living children, demonstrated a significantly lower chance of having zero utilization of maternal care services. This finding was in agreement with a study conducted in Ethiopia [25] that revealed women with five or more living children were three folds more likely to complete the standard maternal continuum of care. This might be due to higher parity may be positively associated with a woman’s likelihood to engage in and complete essential maternal health services, possibly due to increased awareness or experience with healthcare systems gained through prior pregnancies. As women gain more experience with childbirth, they may become increasingly aware of the importance of maternal healthcare services.

Consistent with other studies [24,26,27], our findings showed a negative relationship between women’s educational attainment and zero maternal continuum of care. Women who had secondary or higher education were less inclined to experience zero utilization of maternal continuum of care. Another study conducted in Ethiopia [12] also reported a comparable result of higher zero maternal continuum of care utilization in uneducated mothers. Educated women are often more aware of the importance of maternal health services and can better understand the potential benefits of regular antenatal, delivery, and postnatal care. This awareness enables them to make informed decisions about their health, seek preventive care, and navigate healthcare systems more effectively. Another possible explanation could be educated women are more likely to advocate for themselves within healthcare settings and may feel more empowered to overcome traditional and social barriers to accessing care. This positive association between education and health-seeking behavior highlights the importance of investing in female education as a strategy to improve maternal and child health outcomes.

Women from rich households, demonstrated a lower possibility for zero maternal continuum of care. This finding was consistent with findings in studies conducted in Pakistan [28], Nepal [29], India [30], Rwanda [31], and systematic review and meta-analysis of nine African countries including Ethiopia [2]. This relationship may be attributed to these women’s relatively better access to healthcare facilities and higher levels of health literacy compared to those from lower-income backgrounds, enabling them to seek and utilize maternal health services more effectively. Additionally, marital status appears to play a significant role in maternal healthcare utilization with those who are married have lower likelihood of zero maternal continuum of care. This finding was documented in Rwanda, as married women had higher likelihood of having maternal continuum of care as compared to not-married women [31]. Comparable findings also found in Ethiopia [32,33], East Africa [34], and sub-Saharan African countries [35] that reported higher utilization of ANC, institutional delivery by skilled birth attendant, and PNC services in married women. This might be due to the fact that married women are often more likely to receive social and financial support from their spouses and families, which can positively influence their health-seeking behavior. Spouses may actively support their partners’ health needs, including maternal care, thereby reducing the likelihood of missed or skipped healthcare services. Conversely, unmarried women may experience significant barriers to accessing maternal services as a result of stigma often arises from cultural norms and beliefs that prioritize marriage as a prerequisite for motherhood, leading to negative perceptions of women who are not married but are pregnant or seeking maternal care. Addressing these social barriers is crucial for ensuring that all women, regardless of their marital status, have equal access to essential maternal health services.

Women in the 25–34 age group also had lower odds of zero care, in agreement with a study conducted in sub-Saharan Africa [36]. Likely reflecting greater social and financial stability, which may reduce barriers to accessing services. Women relationship with household head and religion had also influenced zero maternal continuum of care utilization. Daughters of household heads were more likely to have zero maternal care, possibly due to a lack of financial independence or decision-making authority, which can limit healthcare access. Consistent with previous studies in Ethiopia [37,38], protestant women also exhibited a higher likelihood of zero care, potentially reflecting distinct religious or cultural norms that influence health-seeking behavior in some communities.

### Strength and limitation of the study

This study demonstrates several methodological strengths that contribute to its robustness and relevance. By leveraging a machine learning approach, it provides a nuanced analysis of complex, non-linear relationships between various predictors and zero maternal continuum of care utilization, addressing limitations that may arise in traditional statistical methods. The integration of SHapley Additive explanation analysis enhances the interpretability of the machine learning model, allowing a clear understanding of how each predictor contributes to the likelihood of zero maternal care utilization. Additionally, the use of SMOTE addresses class imbalance within the dataset, thereby improving the model’s reliability and its predictive power for minority class outcomes. Moreover, the study is grounded in nationally representative data from the EMDHS, allowing the findings to be generalizable across diverse regions and population groups in Ethiopia.

Despite its strengths, the study has certain limitations. The cross-sectional design of the demographic and health survey (DHS) restricts the ability to draw causal inferences between the identified predictors and zero maternal care utilization, as only associations can be observed. Future research could address these shortcomings by employing longitudinal study designs, which allow for tracking changes and outcomes over time, thereby strengthening the ability to infer causality. In addition, quasi-experimental methods may help to approximate causal relationships in observational settings. Incorporating such approaches in future studies would provide a more robust understanding of the temporal and causal pathways influencing maternal healthcare utilization.

Furthermore, the reliance on secondary data constrained the analysis to the variables available in the survey, potentially excluding other relevant factors such as psychosocial determinants, behavioral, or transportation accessibility that may impact maternal healthcare utilization. The analysis may also overlook region-specific cultural, geographic, or healthcare system factors, particularly, which could influence maternal healthcare access. Lastly, the study’s reliance on survey data may introduce recall bias, as participants might not accurately remember or report their healthcare experiences, particularly regarding the timing and frequency of maternal care visits. This potential for recall bias could affect the accuracy of reported maternal care utilization, especially in cases where services were accessed several months or years prior to the survey.

To enhance the reliability of our model and mitigate the effects of class imbalance, we implemented 10-fold cross-validation and applied the Synthetic Minority Oversampling Technique (SMOTE) during training. These strategies were intended to reduce the risk of overfitting and improve generalizability. However, the possibility of overfitting cannot be entirely excluded, especially given the complexity of ensemble models like LightGBM. While the model exhibited strong discriminatory performance, additional evaluation using calibration plots could provide further insight into the agreement between predicted probabilities and actual outcomes. Moreover, the absence of external validation limits the ability to assess the model’s performance on unseen data. Future research should incorporate external validation and calibration analysis to strengthen confidence in the model’s robustness and applicability in diverse settings.

## Conclusion

In this study, we applied machine learning and SHAP analysis to identify the determinants of zero utilization of the maternal continuum of care among Ethiopian women. Our findings underscore the influence of key socioeconomic, demographic, and regional factors on maternal healthcare access. Women in rural areas, those residing in the Somali region, daughters within households, and Protestant women were more likely to forego maternal healthcare services entirely, suggesting significant disparities in service access and utilization. Conversely, higher educational attainment, marital status, wealth, and increased parity were associated with a decreased likelihood of zero utilization, pointing to factors that may facilitate better access to maternal care.

These insights are crucial for guiding policymakers in developing targeted, context-sensitive interventions that address barriers specific to various demographic and regional groups. To inform policy and interventions effectively, it is essential to prioritize the development of outreach programs that specifically target women in rural and underserved regions, particularly in the Somali region. Additionally, enhancing educational initiatives aimed at increasing awareness of maternal health services and their importance can empower women to seek care. Furthermore, integrating community-based approaches that involve local leaders and healthcare providers can help to build trust and encourage utilization of maternal healthcare services. Future research should focus on evaluating the effectiveness of these interventions and exploring additional factors that may influence maternal healthcare utilization. It’s crucial to foster collaboration between researchers, policymakers, and community stakeholders to make significant strides toward improving maternal health outcomes and achieving equitable healthcare access across Ethiopia.
